# Ethical considerations in AI for child health and recommendations for child-centered medical AI

**DOI:** 10.1038/s41746-025-01541-1

**Published:** 2025-03-10

**Authors:** Seo Yi Chng, Mark Jun Wen Tern, Yung Seng Lee, Lionel Tim-Ee Cheng, Jeevesh Kapur, Johan Gunnar Eriksson, Yap Seng Chong, Julian Savulescu

**Affiliations:** 1 Krsyma Medical AI Pte Ltd, Singapore, Singapore; 2 St Helens and Knowsley NHS Foundation Trust, Merseyside, England; 3 Department of Paediatrics, National University of Singapore, Singapore, Singapore; 4 Department of Diagnostic Radiology, Singapore General Hospital, Singapore, Singapore; 5 Department of Diagnostic Imaging, National University Hospital, Singapore, Singapore; 6 Singapore Institute for Clinical Sciences (SICS), Agency for Science, Technology and Research (A∗ STAR), Singapore, Singapore; 7 Department of Obstetrics & Gynaecology, Yong Loo Lin School of Medicine, National University of Singapore (NUS), Singapore, Singapore; 8 Department of General Practice and Primary Health Care, University of Helsinki and Helsinki University Hospital, Helsinki, Finland; 9 Folkhälsan Research Center, Helsinki, Finland; 10 Centre for Biomedical Ethics, Yong Loo Lin School of Medicine, National University of Singapore, Singapore, Singapore; 11 Biomedical Research Group, Murdoch Children’s Research Institute, Melbourne, VIC Australia; 12 Oxford Uehiro Centre for Practical Ethics, Faculty of Philosophy, University of Oxford, Oxford, UK

**Keywords:** Medical ethics, Paediatrics, Paediatric research, Machine learning, Computational models

## Abstract

There does not exist any previous comprehensive review on AI ethics in child health or any guidelines for management, unlike in adult medicine. This review describes ethical principles in AI for child health and provides recommendations for child-centered medical AI. We also introduce the Pediatrics EthicAl Recommendations List for AI (PEARL-AI) framework for clinicians and AI developers to ensure ethical AI enabled systems in healthcare for children.

## Introduction

Children are not miniature versions of adults, as children undergo age-associated changes in organ function and neurodevelopment^1,2^. Even within the pediatric age group of 0–18 years, there is a large disparity between preterm neonates with immaturely developed organs, compared to post-pubertal adolescents with adult physiology^1,3^.

Artificial Intelligence (AI) is playing an increasingly important role in healthcare. In pediatrics, AI is used in a wide variety of fields, such as in radiology for the diagnosis of developmental dysplasia of the hip^4^ and in genetics for the diagnosis of rare diseases^5^. AI holds much promise for improving the healthcare of children worldwide, including in less developed and underprivileged communities with limited access to specialist pediatricians.

There is widespread awareness of the importance of AI ethics and governance for adults, but less emphasis has been placed on AI ethics and governance for children. This review article aims to describe ethical principles and challenges in the use of AI in healthcare for children. Important ethical principles that will be covered include non-maleficence, beneficence, autonomy, justice and transferability, transparency and explainability, privacy, dependability, auditability, knowledge management, accountability, and trust. The final section in this article will provide recommendations for child-centered medical AI.

## Methodology

A literature search in PubMed for relevant articles related to AI ethics in child health was conducted in January 2024 and repeated in September 2024. We conducted a search using “Artificial Intelligence or AI” and “Ethics” as search terms and “Guidelines”, “Practice Guidelines”, “Review”, and “Systematic Review” as the article type. The filters for “Age: Child: birth-18 years” and “Article Language: English” were also applied. The abstracts in the returned search were reviewed for articles that either discussed AI ethics in child health or provided recommendations and guidelines on ensuring ethical AI in child health. Articles that fulfilled the above criteria were selected to have their full text reviewed. The lists of references from the selected articles were also screened to obtain further relevant articles.

A literature search for relevant articles related to AI ethics in children was similarly conducted in January 2024 using the Google search engine. We conducted a search using “AI ethics in children” as the search phrase and obtained the first 40 results returned. The webpages in the returned search were reviewed for articles that either discussed AI ethics in children or provided recommendations and guidelines on ensuring ethical AI in children. Articles that fulfilled the above criteria were selected to have their full text reviewed. The lists of references from the selected articles were also screened to obtain further relevant articles.

A search was also performed for policy documents or position statements on the websites of organizations that were deemed relevant to our review. These included UNICEF (on children), WHO (on health), International Pediatric Association, American Academy of Pediatrics, Royal College of Paediatrics and Child Health (on children’s health), International Medical Informatics Association and American Medical Informatics Association (on medical informatics). Policy documents or position statements that fulfilled the above criteria were selected to have their full text reviewed. The lists of references from the selected documents were also screened to obtain further relevant articles.

Our search strategy revealed that there does not exist any previous comprehensive review or framework on AI ethical issues in child health, nor are there any guidelines for management, unlike in adult medicine^6,7^. There is a published 2-page review with framework based on only 7 references on the ethics of AI in Pediatrics, focusing mainly on the use of generative AI chatbots that utilize Large Language Models^8^.

There are, however publications that address AI ethical issues in subspecialty pediatric medicine. These include embryology^9^, neonatology^10^, genomic medicine^5^, and radiology^11,12^.

There are several guidelines on the ethics regarding the use of AI in children^13–16^, but these are not specific to the practice of Medicine.

## Ethical considerations

First described in 1979, Beauchamp and Childress’s landmark work^17^ on foundational principles of medical ethics is ever more important in considering the ethical debate surrounding AI-enabled applications and usage. Key principles then highlighted include—autonomy, beneficence, non-maleficence, and justice, which have been cornerstones of ethical discussions in healthcare. Jobin et al. identified other ethical concerns with regard to AI^18^, and these concerns include transparency, privacy, and trust. The American Medical Informatics Association (AMIA) has also defined additional AI principles that include dependability, auditability, knowledge management and accountability^7^. Unfortunately, some of these ethical principles may conflict with one another, such as justice and privacy, as illustrated below.

### Non-maleficence

Non-maleficence implies the need for AI to be safe and not to cause harm^18,19^. References to non-maleficence in AI ethics occur more commonly than beneficence^18^, likely due to society’s concerns that AI may intentionally or unintentionally inflict harm. Prioritizing non-maleficence before beneficence when approaching AI systems by no means suggests that AI systems are fraught with risks or harm. Rather, it highlights the approach to ethical issues in the context of AI. Before an AI system is implemented for child health, there must exist convincing evidence that it results in no harm or that benefits can be confidently expected to outweigh harm, notwithstanding any benefits that it can bring to the children. Evidence-based health informatics (EBHI) supports the use of concrete scientific evidence in decision-making regarding the implementation of technological healthcare systems^20^.

In embryology, the process of in-vitro fertilization involves selecting the best embryo for transfer. Ethical principles guide the selection of one embryo over another. The ‘best’ embryo has the highest potential to result in a viable pregnancy, whilst preventing the birth of children with conditions that would shorten their lifespan or significantly decrease their quality of life^9^. AI has been used to rank embryos using images and time-lapsed videos as input^21^. AI has also been used in pre-implantation genetic screening of embryos non-invasively without the need for an embryo biopsy^22^. In 2019, scientists in a fertility clinic in Australia developed a non-invasive test (that did not use AI) for preimplantation genetic screening of embryos^23,24^, and introduced it prematurely for clinical use^9^. There was a marked discrepancy in results between validation studies and real-world clinical experience^25^. Importantly and significantly, embryos erroneously deemed genetically abnormal by the novel test and unsuitable for transfer appear to have been discarded^26^, resulting in a class action suit in Australia^27^. Although the non-invasive test above did not utilize AI, it nevertheless serves as a cautionary tale. Experts have argued that prioritizing embryos for transfer using novel technologies, such as AI, is acceptable^9^, but discarding embryos based on unproven advances is not^9^; thereby emphasizing the need for caution and a balanced approach to ensure that the benefits of novel technologies outweigh any potential harm.

AI might deepen existing moral controversies. For example, coupled with whole genome or exome sequencing, AI could facilitate massive genomic examination of embryos for novel disorders, dispositions or polygenic risk of disease or non-disease traits (such as intelligence). This would move beyond targeted preimplantation genetic diagnosis to massive prenatal “screening” raising significant ethical issues, even facilitating polygenic editing.

AI systems used in healthcare are often designed to include a “human-in-the-loop”. The prediction made by the AI system is checked by a human expert, such that the AI augments but does not automate decision-making. The knowledge, skills, experience and judgment of the healthcare professional are important in case- contextualization, as no case is “standard” and each child comes with his own unique medical, family and social history. Although having a human in the loop decreases the risk of an AI causing harm, there is a risk of introducing human bias and decreasing justice and fairness. AI-enabled decisions are more objective and reproducible unless the source training data was biased or derived from a disparate population from which it is being used.

AI systems that are used outside of healthcare settings can also have an impact on children’s health. Social media and streaming platforms are changing how children interact with content. With touchscreen technology and intuitive user interfaces, even very young children can access these applications with ease^28^. AI recommendation algorithms are optimized to keep children engaged on the platform for extended periods rather than to prioritize content quality^29^. There have been multiple studies that have highlighted the adverse effect of prolonged screen time on the cognitive development and neurobehavioral development of children^30,31^, and on the development of obesity^32^, and its related complications. Excessive screen time is positively associated with behavioral and conduct problems, developmental delay, speech disorder, learning disability, autism spectrum disorders and attention deficit hyperactivity disorder, especially for preschoolers and boys, and the dose-response relationships are significant^30^.

### Beneficence

Beneficence or promoting good can be seen as benefiting an individual or a group of persons collectively^18^. AI must benefit all children, including children from different ages, ethnicities, geographical regions and socioeconomic conditions. These include the most marginalized children and children from minority groups.

In healthcare, AI has demonstrated its ability to benefit the care of sick children in out-patient^33,34^ and in-patient care^35^. In genomics, AI has been used in both prenatal and pediatric settings. AI can use genotypes to predict phenotypes (genotype-to-phenotype) and can also use phenotypes to predict genotypes (phenotype-to-genotype). Identifai Genetics can determine in the first trimester of pregnancy whether there is a higher chance a baby will be born with any genetic disorder, using cell-free fetal DNA circulating in the maternal blood^33^, allowing in-utero treatment of some genetic diseases. Face2Gene uses deep learning and computer vision to convert patient images into de-identified mathematical facial descriptors^36,37^. The patient’s facial descriptors are compared to syndrome gestalts to quantify similarity (gestalt scores) to generate a prioritized list of syndromic diagnosis^36,37^. Face2Gene supports over 7000 genetic disorders^34^, and is routinely used in clinical practice by geneticists.

An AI platform combining genomic sequencing with automated phenotyping using natural language processing prospectively diagnosed three critically ill infants in intensive care with a mean time saving of 22 h, and the early diagnosis impacted treatment in each case^35^. In these time-critical scenarios, rapid diagnosis by AI can have a meaningful impact to improve clinical outcomes for these seriously ill children with rare genetic diseases. It also allows transfer to palliative care and avoidance of invasive procedures for diagnoses that are incompatible with life.

### Autonomy

Autonomy can be viewed as positive freedom or negative freedom^18^. Positive freedom is seen as the ability for self-determination^38^, whereas negative freedom is the ability to be free from interference, such as from technological experimentation^39^ or surveillance^40^.

Unlike adults, who are able to consent, a parent or legal guardian must provide consent for the collection of a child’s medical data or the use of an AI-enabled device in a child. Decisionally competent adolescents have developing autonomy, and their consent should be sought, as well as that of parents. Gillick competence can be applied when determining whether a child under 16 is competent to consent^41,42^. Gillick competence is dependent on the child’s maturity and intelligence, and higher levels of competence are required for more complicated decisions. Consent obtained from a Gillick-competent child cannot be overruled by the child’s parents. However, when a Gillick competent child refuses consent, the consent can be obtained from the child’s parent or guardian. In accordance with the United Nations Convention on the Rights of the Child, every child has the right to be informed and to express their views freely regarding matters relevant to them, and these views should be considered in accordance with the child’s maturity^43^. Although a younger child is not legally able to give consent, the child has the freedom to assent or dissent after being informed in age-appropriate language^44^.

The use of AI in pediatric care should not infringe the child’s right to an open future^45^. This can occur through infringements of confidentiality and privacy, or generally if decisions are made on the basis of AI which unreasonably narrows the child’s future options.

### Justice and transferability

Justice is defined as fairness in terms of access to AI^18,19^, data^18^, and the benefits of AI^18,46^; and the prevention of bias^18,19^, and discrimination^18,19^. Justice encompasses equity for all, including vulnerable groups such as minority groups, mothers-to-be and children. AI must benefit all children, including children from different ages, ethnicities, geographical regions and socioeconomic conditions.

Underprivileged communities, including their children, are similarly disadvantaged in the digital world^47^. Technology (including AI) may increase inequality in under-resourced, less-connected communities^48^ due to limited access to technology and lower digital literacy. This impacts the ability of the healthcare teams in these communities to leverage on AI in both adult and pediatric medicine. Moreover, machine learning algorithms trained on pediatric data from developed countries may not be applicable to children in less developed countries, resulting in incorrect predictions. These AI applications that were trained on non-representative populations can potentially perpetuate rather than reduce bias^49^. AI systems risk compromising children’s right to equitable access to the highest attainable standard of healthcare^43^.

However, AI can also promote equality by connecting under-developed communities to developed communities. The Pediatric Moonshot project was launched in 2020 in an effort to reduce healthcare inequity, lower cost and improve outcomes for children globally^50^. The Pediatric Moonshot project aims to link all the children’s hospitals in the world on the cloud by creating privacy-preserving real-time AI applications based on access to data. Edge zones have been deployed in 3 continents (North America, South America, and Europe). There is a shortage of specialist pediatricians in underdeveloped countries, and the Pediatric Moonshot project includes Mercury, a global image-sharing network to allow non-children’s hospitals or clinics to share images with pediatricians in children’s hospitals for expert opinion. The Pediatric Moonshot project also includes Gemini, an AI research lab for children, designed to pioneer privacy-preserving, de-centralized training of AI applications in child health that can also be deployed on mobile devices for use by doctors serving under-privileged communities.

Algorithmic bias is the systemic under or over-prediction of probabilities for a specific population, such as children. Fairness (unbiasedness) is multifaceted, has many different definitions and can be measured by various metrics^51^. Fairness metrics used for AI models in healthcare include well-calibration, balance for positive class, and balance for negative class. It is important to note that these 3 conditions for fairness cannot typically be achieved at the same time by an AI model, except under very specific conditions^52^. Hence, there is no universal one-size-fits-all definition of fairness, and some definitions are incompatible with others. The appropriate definition and metric of fairness used largely depends on the healthcare context.

Al-enabled devices that were trained on adult data only may underperform when used in children. Several studies have investigated the use of adult AI in pediatric patients and results have highlighted difficulties in generalizing AI across the age spectrum^53–57^. For example, AI developed to detect vertebral fractures in adults was unreliable in children with a low sensitivity of 36% for the detection of mild vertebral fractures^54^. A deep learning algorithm, EchoNet-Peds, that was trained on pediatric echocardiograms performed significantly better to estimate ejection fraction than an adult model applied to the same data^55^. As pediatric care is commonly undertaken in facilities that manage both adults and children, AI-enabled devices not evaluated in children could unwittingly be used by healthcare providers on children, resulting in adverse outcomes. Thus far, most AI-driven radiology solutions have been designed for adult patients. Of late, radiology imaging advocacy groups have appealed to the US Congress to create policies that address the lack of AI-based innovations tailored specifically for pediatric care^58^.

As such, it is important to consider the transferability of AI systems to the context of pediatric healthcare. Transferability is a measure for how effective a health intervention, initially evaluated and validated in one context, can be applied to another^59^. AI models are prone to systemic bias arising from the training data, which limits the range of application. Even if the training data originates from a diverse population, the differences in quantity can greatly skew outputs. Children from diverse backgrounds may experience vastly different health challenges, which can be due to factors such as demographic characteristics, upbringing, culture, access to healthcare services, and their surrounding environment. Failure to account for these differences could lead to bias and disparities in the quality of care, disproportionately affecting vulnerable children.

### Transparency and explainability

Transparency includes both technological transparency and organizational transparency. Technological transparency refers to the communication and disclosure to stakeholders of the use of AI^18,19^, including to the healthcare team, pediatric patients, and their parents, or guardians. Parents value transparency, and disclosure pathways should be developed to support this expectation^60^. Transparency also refers to efforts to increase explainability and interpretability of AI-enabled devices^18^.

Organizational transparency refers to the disclosure to patients and parents of conflicts of interest. It is not uncommon for AI-enabled mobile health applications to have both a diagnostic and a therapeutic arm, wherein a diagnosis made is followed by a redirection of the user to an e-commerce platform with therapeutic products, such as in esthetic medicine websites that are also used by adolescents. Appropriate disclosure of any conflicts of interest between the developer of the AI diagnostic app and the manufacturer of the recommended therapeutic products is frequently absent.

Transparency is seen as a key enabler of the various ethical principles. Only with transparency and understanding, can there be nonmaleficence, autonomy^18^, and trust^46,61–63^.

### Privacy

Privacy relates to the need for data protection and data security^18^. While privacy is a right for all children as per the UN Convention on the Rights of the Child^43^, there is marked variability in adolescent privacy laws not only between countries, but also between states within the same country for consent and privacy regarding substance abuse, mental health, contraception, human immunodeficiency virus infection, and other sexually transmitted infections^64^. This creates challenges for AI developers looking to build AI systems for the above health conditions for older children.

Fitness trackers and wearables, and digital health apps such as menstruation tracking, sleep tracking, and mental health apps, are popular among adolescents^65^. These commercial apps collect sensitive data, including real-time geolocation data and reported or inferred emotional states^65^. As mobile phone apps collect a large amount of identifying data, it is almost impossible to de-identify data in order to protect privacy^66^. Stigma and discrimination can result from leakage of sensitive health data, and while this negatively affects patients of all ages, the vulnerability and young age of children means that any inadvertent disclosure of such data would have longer-lasting effects in children^65^.

De-identified data is typically used to train AI systems. However, there is a real possibility of de-identified pediatric data to be re-identified, particularly for children with rare genetic diseases, thereby resulting in an infringement of privacy and possible harm. Larger datasets, which include data from pediatric patients, are needed for the unbiased training of AI-enabled devices used by children.

Unfortunately, this may result in not only the loss of autonomy, but also the possibility of re-identification and loss of privacy for certain children and their families.

### Dependability

Dependability refers to the need for AI systems to be robust, secure and resilient, where in the event of a malfunction, the system must ensure that it does not put the patient or the clinical setting in an unsafe state^7^. This principle is especially important for pediatric patients, as they may be less capable of voicing concerns or understanding risks and less likely to be aware when an adverse event has occurred compared to adults. Without proper supervision, such malfunctions can be catastrophic.

### Auditability

Auditability is the requirement for any capable AI system to document its decision-making process via an “audit trail” which captures input and output values as well as changes in performances and model states^7^. This is a layer of transparency that is critical for understanding how the model functions and evolves over time. In pediatric care, this allows clinicians to ensure that recommendations made by an AI-enabled system align with the needs of children and identify any systemic error that may disproportionately affect them. The audit log is also important for clinicians to evaluate changes within the system over time. For medical-legal purposes, the audit trail for AI in pediatric patients may need to be retained until the age of maturity (18 years) plus an additional 3 years (21 years)^7^.

### Knowledge management

Children’s health can be significantly impacted by a wide range of factors, from genetic to environmental. In the present day, these factors can fluctuate widely within short periods of time, and vary among children. AI models for pediatric healthcare, as a result, may become outdated and less effective as time goes on.

### Accountability

Accountability is the requirement for organizations responsible for creating, deploying and maintaining the AI system to actively supervise its usage and address any concerns raised^7^. As we have mentioned above, children represent a specially vulnerable population who may be unaware of the potential risks from AI systems. It is then up to parents and clinicians to voice concerns regarding the safety of the child. Accountability ensures that any potential failures in AI systems do not disproportionately burden individual clinicians but are addressed in a way that protects both healthcare providers and the children under their care.

Accountability also encompasses professional liability. The clinician in charge of the patient is potentially liable for any harm from use of the AI-enabled system on his pediatric patients, and his professional license is at risk. In future, the clinician could also be held accountable for his or her failure to utilize AI-enabled systems on his patients if this becomes the standard of care.

### Trust

Trust refers to trustworthy AI and is a byproduct of the above ethical principles. It is generally recognized that trust is needed for AI adoption and for AI to fulfill its potential for good. Conversely, it can be argued that trust is the one ethical principle in which we should not have 100% of, in that we should never place complete trust in an AI-enabled medical device.

## Recommendations for child-centric medical AI

At present, none of the professional bodies for child health (including the International Pediatric Association representing pediatricians from over 144 countries in over 176 member societies, the American Academy of Pediatrics, and the Royal College of Paediatrics and Child Health in the United Kingdom) have published a set of guidelines or recommendations for child-centered medical AI. Similarly, none of the medical informatics associations (including the International Medical Informatics Association and the American Medical Informatics Association) have published guidelines or recommendations for pediatric medical AI. What is currently available are 1) guidelines for AI ethics and governance in adult medicine^6,7^ and 2) policy documents from United Nations Children’s Fund (UNICEF) and the like on AI ethics and governance pertaining to children^13–16^ but not specific to child health.

In this review paper, we based our recommendations for child-centered AI on the policy guidance by UNICEF^13^, and we elaborated on these recommendations in the context of child health.

The overarching recommendations by UNICEF are to develop and deploy AI systems in a manner that upholds children’s collective rights to protection, provision and participation whilst nurturing various stakeholders and adapting to the national or local context^13^. UNICEF has specific recommendations that are discussed below.

### Ensure AI used in healthcare promotes children’s development and wellbeing

UNICEF recognizes that AI systems can support the realization of every child’s right to good health and to flourish across mental, physical, social, and environmental spheres of life^13^. UNICEF recommends prioritizing how AI systems can benefit children and to leverage AI to support children’s well-being^13^. AI design should adopt a child-centered approach, which should include safety-by-design, privacy-by-design and inclusion-by-design.

### Ensure inclusion of and for children during the design and development of healthcare AI

All of the four ethical principles (respect for autonomy, non-maleficence, beneficence, and justice) require high-quality evidence, This includes AI. There must be an inclusive design approach when developing AI products that will be used by children or impact them, and there should be meaningful child participation, both in AI policies and in the design and development processes^13^. Ideally, this should include randomized controlled trials of the use of AI in children where feasible.

Within the health care context, conducting clinical trials in children is challenging due to the heterogeneity of the subjects^1^ and ethical concerns resulting in strict laws and ethical guidelines^67,68^. In addition, children are collectively a smaller population than adults, and children have fewer chronic diseases, making it less financially attractive for commercial vendors to develop AI-enabled devices for children. Notwithstanding the difficulties, the National Institute of Health states that “children (i.e., individuals under the age of 18) must be included in all human subjects research, conducted or supported by the NIH, unless there are scientific and ethical reasons not to include them”^69,70^. The NIH policy was developed because “medical treatments applied to children are often based upon testing done only in adults, and scientifically evaluated treatments are less available to children”^69^. Specifically in the context of AI systems, the White Paper by the American College of Radiology (ACR) recommends the inclusion of pediatric patients in AI models that are developed and potentially applicable to children^11^. Developers could be incentivized to develop AI using suitable pediatric data, resulting either in separate pediatric models or in combined adult and pediatric models. The ACR also recommends the incorporation of AI into clinical practice guidelines for children when appropriate^11^.

### Ensure AI used in healthcare for children prioritize fairness, non-discrimination and equitable access

The most marginalized children, including children from minority groups, should be supported so that they may benefit from AI systems. Datasets should include a diversity of children’s data, including children from different regions, ages, socioeconomic conditions, and ethnicities, in order to remove prejudicial bias against children or against certain groups of children that results in discrimination and exclusion^13^.

The American Medical Informatics Association Position Paper states that “AI must be subject to increased scrutiny when applied to vulnerable groups including children, particularly in cases where such groups were under-represented in the data used to train the AI^7^.” All AI healthcare models used in children should be tested for fairness using an appropriate definition of fairness and a suitable metric of fairness that caters to the specific context. For AI-enabled devices that are used by both adults and children, the model must not be biased against children. For AI-enabled devices that are exclusively designed for children, the model must not discriminate against any group of children (such as by race, geographical location, or socioeconomic situation). Given the known limitations in conducting clinical trials with children, we may sometimes have to argue for a benign form of discrimination in favor of children.

ACCEPT-AI is a framework designed to evaluate AI studies that include pediatric populations and can be used to check for age-related algorithmic bias throughout the AI life cycle, from study design to post-deployment^71^. If needed, pre-processing, in-processing, and/or post-processing can be implemented to mitigate bias. Bias should be minimized as far as possible, but it is not usually possible to totally eliminate bias.

However, ensuring fairness in AI for children goes beyond addressing algorithmic bias. Economic and organizational values must also be taken into account to ensure equitable access to AI-driven healthcare systems for all children, regardless of socioeconomic status. These developments should aim to provide better healthcare outcomes for children using fewer resources, and AI system providers should aim to provide business models that offer more value to users^72^. This ensures that new healthcare systems remain accessible to lower-income populations and reduces the burden on healthcare providers in under-developed communities. In this sense, AI developments must be inclusive, engaging a broad range of stakeholders to ensure that the perspectives of children, caregivers, healthcare providers, policymakers, and communities are incorporated into the design and deployment processes^73^. Inclusivity helps mitigate the risk of further marginalizing vulnerable populations and ensures that the benefits of AI can be equitably distributed across diverse groups of children.

### Ensure AI enabled healthcare systems protect children’s data and privacy

There must be a responsible data approach to the handling of children’s data^65^. A balance must be found such that there is sufficient data about children for the development of AI systems while minimizing data collection to safeguard privacy and security^65^. AI systems should adopt a privacy-by-design approach. Not only is there a need to protect an individual child’s right to privacy, but there is also a need to protect collective groups of children (such as a racial group) to prevent profiling^13^.

UNICEF promotes children maintaining control over their own data with the capacity to access, securely share, understand the use of, and delete their data, in accordance with their age and maturity^65^. However, parents and guardians need to provide consent for the use of younger children’s data. Furthermore, as children’s understanding develops with age, the consent process should be revisited periodically as the child grows^13^. As children mature and attain the age of consent, they can reverse the consent previously provided by their parent or legal guardian and exercise their ‘right to be forgotten’ and for their data to be erased^74^.

### Ensure safety for children when AI is used in healthcare

With respect to how AI systems (including AI-enabled mobile health applications) interact with users, children should not be exposed to content targeting that could harm their mental or physical health. Additionally, in keeping with online safety recommendations, children and their parents should have access to child safety tools. These tools should include options to control the content children are exposed to, limit the public visibility of profile information, restrict other users from contacting or interacting with an account used by a child, and manage location sharing^75^.

UNICEF advocates continuously assessing and monitoring AI’s impact on children throughout the entire AI development life cycle and testing AI systems for safety, security, and robustness^13^. AI systems used in healthcare, in particular those used for children, should have appropriate human agency, oversight and control measures with humans in the loop as far as possible.

In Pediatric Medicine, off-label use of medication is common^76^, as there are fewer legalized medicines and dosage forms for the pediatric population^77^. Legal restrictions on the conduct of clinical trials in children exacerbate the lag in the regulation of medicines for pediatric use^78,79^. Off-label use is associated with increased uncertainty on efficacy and increased risk for adverse effects. Significantly, more off-label medicines are prescribed in the neonatal and pediatric intensive care units^76,80^, and this may reflect the dire do-or-die situation that makes off-label drug use less of an issue for clinicians. With regards to off-label use of drugs and medical devices in the United States, once a drug or device receives regulatory approval, physicians can exercise professional judgment and legally prescribe the drug or device for any indication they deem safe and effective, irrespective of official FDA-approved indications^81^. The American Academy of Pediatrics (AAP) Policy Statement on the Off-Label Use of Medical Devices in Children states that “The clinical need for devices to diagnose and treat diseases or conditions occurring in children has led to the widespread and necessary practice in pediatric medicine and surgery of using approved devices for off-label or physician- directed applications that are not included in FDA-approved labeling. This practice is common and often appropriate, even with the highest-risk (class III) devices.”^82^ The FDA Guidance document on “Off-Label and Investigational Use Of Marketed Drugs, Biologics, and Medical Devices” states that “If physicians use a product for an indication not in the approved labeling, they have the responsibility to be well informed about the product, to base its use on firm scientific rationale and on sound medical evidence.”^83^ The AAP Policy Statement on the Off-Label Use of Drugs in Children states that “Off-label use is neither incorrect nor investigational if based on sound scientific evidence, expert medical judgment, or published literature.”^84^ The emphasis on scientific evidence and published literature avoids experimental and potentially unsafe practices.

Drawing from the experience of off-label drug and device use, with the added knowledge that AI systems behave unpredictably when applied to patients demographically different from their training population, there is an urgent need for additional research and recommendations by key opinion leaders on the risks and benefits of off-label use of AI-enabled devices in pediatric patients and to set new standards of evidence before AI is deployed on children. Robust informatics evaluation frameworks are also crucial when developing AI systems for pediatric care to ensure that the design prioritizes ethics and equity, assessing limitations and risks while also helping users understand system logics^85^. Evidence-based health informatics requires the need for concrete scientific evidence in assessing performances and risks associated with AI systems^20^.

Moreover, AI models must be also continuously updated and retrained to account for new pediatric data^7^, reflecting such changes to ensure that the outputs from the models remain accurate, reproducible, and relevant. This prevents degradations in model effectiveness from causing harm to children. Developers must clearly document when AI models are created, revalidated, and set to expire^7^.

Aside from this, AI systems need to adopt a fail-safe design and be thoroughly tested for robustness to ensure that their performance does not degrade in unforeseen circumstances^7^. This prevents potential malfunctions from compromising the safety of the child.

AI-enabled systems must have cybersecurity measures in place to protect against unauthorized access, modification and disruption, whilst maintaining confidentiality, integrity and availability. These protective mechanisms must secure both the AI system as well as the data of the children.

### Ensure that AI in healthcare supports transparency, explainability and accountability for children

Medical professionals should be informed about the use of an AI-enabled device and the limitations of the AI system including inclusion and exclusion criteria. Parents and children who interact with an AI medical system also have the right to be informed using age-appropriate language and an inclusive manner, to understand how the system works and how it uses and maintains confidential data^13^. AI systems should be developed so that children are protected and empowered by legal and regulatory frameworks, irrespective of children’s vulnerability and understanding of the AI system^13^. These AI governance frameworks must be regularly reviewed and updated to protect the rights of children. AI regulatory bodies must be established to continually monitor and correct any ethical infringements to the rights of children.

Physicians should determine prior to the use of any AI system whether the device has been specifically evaluated in pediatric patients by referring to the indications for use section and the 510(k) summary in the FDA 510(k) database^12^. Alternatively, physicians can refer to the user manual or ask for such information from the device vendor^11^. The American College of Radiology (ACR) has recommended including a “statement regarding authorization specifically for use in children, including a description of the evidence that does/does not support use in children, or if there is a lack of such evidence” on all FDA-authorized AI devices^86^. The ACR also recommended a highly visible nutrition label-style summary of AI-enabled device information that could include a pediatric use section^86^. The above would guide decision-making related to AI device acquisition, implementation, and appropriate use^11,86^ and facilitate properly informed consent. If the AI-enabled device has not been evaluated in children, physicians should exercise caution and clinical judgment and disclose this to parents and competent children.

As far as possible, developers of AI should use interpretable and not black-box AI for building AI systems for children. This is especially important for irreversible decisions with far-reaching consequences, such as for embryo selection in in-vitro fertilization treatment^87^, or for predicting futility of care in critically ill neonates and children. Developers should also provide accessible mechanisms for reporting and escalating concerns regarding the AI system to medical professionals and families, ensuring that risks are promptly assessed and mitigated, and that complaints are properly addressed. Redress should be offered in case of harm^7^.

### Empower governments and businesses with knowledge of AI and children’s rights

Policymakers, management, and AI system developers must have awareness and knowledge of AI and children’s rights, and be committed to child-centered AI and translating this into practice^13^.

### Support governments and businesses in creating an enabling environment for child-centered medical AI

Governments and businesses should invest in infrastructure development to address the digital divide and aim for equitable sharing of the benefits of AI^13^. Not only must funding and incentives be provided for child-centered AI policies and strategies, support must be provided for rigorous research on AI for and with children across the AI system’s life cycle^13^.

The United Nations Secretary-General’s High-level Panel on Digital Cooperation recommends increasing international cooperation on AI by investment in open source software, open data, open AI models, open standards, and open content^88^. Child-centered AI systems would greatly benefit from government and private sector cooperation and from the sharing of resources and approaches^13^.

**Fig. 1:**
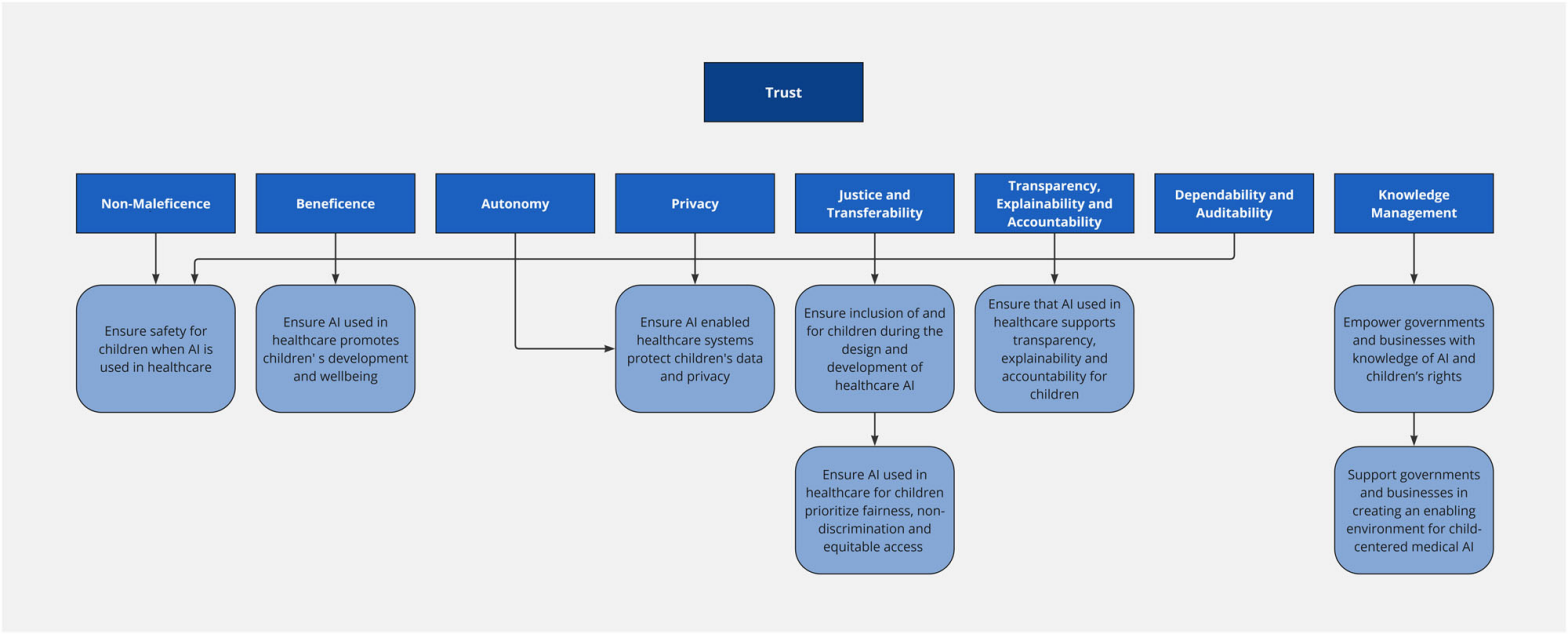
Linking of key ethical concepts and recommendations for child-centered medical AI. The key ethical considerations in AI for child health are non-maleficience, beneficence, autonomy, privacy, justice and transferability, transparency and explainability, accountability, dependability, auditability and knowledge management. Only when these ethical principles are upheld, will there be trust in the AI-enabled system. These ethical principles are linked to recommendations for action to support child-centered medical AI.

The key concepts linking the ethical principles and recommendations for child-centered medical AI are summarized in Fig. 1.

## PEARL-AI framework

From our comprehensive review of ethical principles in AI for child health and the recommendations we have collated for advancing child-centered medical AI, we present the Pediatrics EthicAl Recommendations List for AI (PEARL-AI) framework (Table 1). As there is an absence of both randomized controlled trials and large non-randomized trials in the use of AI in healthcare for children, all the recommendations in the PEARL-AI framework are built using Level C quality of evidence based on previously published opinions of experts. In the framework, we have also included new recommendations that we believe to be important in ethical AI for pediatric healthcare, for which we have elaborated on in the previous section on recommendations for child-centric medical AI.

**Table 1:** PEARL-AI framework for clinicians, academics, administrators and developers

Recommendation	Action	Fulfillment (Complete/Partial/Absent/Not Applicable) with Elaboration
Ensure AI used in healthcare promotes children’s development and wellbeing	AI development/implementation prioritizes benefitting children and supporting children’s well-being^11,13^	
	There is no or minimal potential risk of harm to children from use of the AI-enabled system^7,13^	
Ensure safety for children when AI is used in healthcare	The AI-enabled system used for pediatric healthcare has appropriate human agency, oversight and control measures with a human in the loop as far as possible	
	A fail-safe design is adopted to assure that the performance of the AI-enabled system does not degrade in unforeseen circumstances^7^	
	There is thorough testing for accuracy, safety, security, reproducibility and robustness before the AI-enabled system is deployed on children^13^	
	There is a process to continuously assess and monitor the impact of the AI-enabled system on children^13,86^	
	There is clear documentation as to when AI models are created, validated, and set to expire^7^	
	The AI-enabled system is secure from unauthorized access, modification and disruption whilst maintaining confidentiality, integrity and availability	
Ensure inclusion of and for children during the design and development of healthcare AI	There is meaningful child participation in AI policies and in the design and development processes^11,13,73^	
	AI development utilizes randomized controlled trials in children where feasible	
Ensure AI used in healthcare for children prioritize fairness, non-discrimination and equitable access	Datasets include children as subjects^11,69,70^	
	Datasets are not biased against certain groups of children based on age, ethnicity, region or socioeconomic status^7,13^	
	Tests for fairness (in children and in particular sub-groups of children) using suitable metrics are performed	
	There is equitable access to the AI-enabled system for all children, regardless of socioeconomic status^7,11,13^	
Ensure AI-enabled healthcare systems protect children’s data and privacy	Data collection is minimized with only necessary data collected^65^	
	Consent is required from parents and guardians for the collection, use or storage of younger children’s data^65^	
	Children are able to access, understand the use of, and delete their data in accordance with maturity^43,44,65^	
	Children who reach the age of consent are able to reverse parental consent previously given^74^	
Ensure that AI in healthcare supports transparency, explainability and accountability for children	Medical professionals, parents and children are informed about the use of an AI-enabled system, how the AI-enabled system makes its decisions, and its limitations^11,13,60,86^	
	Parents and children are informed regarding how the AI-enabled system uses and maintains confidential data^13^	
	The AI-enabled system includes a statement on authorizing its use in children, including evidence that supports use in children or a lack thereof^11,86^	
	Developers use interpretable and not black-box AI for building AI-enabled systems for children, in particular for irreversible decisions with far reaching consequences^6,87^	
	Developers and manufacturers provide accessible mechanisms to medical professionals and families for reporting and escalating concerns on the AI-enabled system^7,11,86^	

This framework is intended as a practical, actionable resource for clinicians, academics, healthcare administrators, and AI developers. The PEARL-AI framework will be regularly updated to reflect new evidence and developments in the field of AI in healthcare in children, to ensure that AI-enabled systems in healthcare uphold the highest standards of ethics while addressing the unique needs and vulnerabilities of children.

### A systematic child-centric approach

The PEARL-AI framework is designed to be a child-centric and structured guide that supports ethical decision-making throughout all phases of the AI lifecycle. By placing children at the core of its considerations, the framework prioritizes child health, rights, and well-being in every stage of AI development and deployment.

The ethical challenges inherent in AI development are magnified for pediatric populations due to their vulnerability, dependency on caregivers, and limited ability to advocate for themselves. This makes the implementation of a framework like PEARL-AI essential.

### Proactive ethical oversight

The PEARL-AI framework supports proactive ethical oversight for identifying and addressing potential ethical breaches early in the AI development process. Key features of the framework include:

1. Child-Centered Safeguards: Recommendations for designing algorithms and interfaces that are sensitive to the unique physical, cognitive, and emotional needs of children.
2. Ethical Risk Assessment: A structured evaluation of potential risks to children’s well-being posed by AI models, including bias, discrimination, and unintended outcomes.
3. Stakeholder Engagement: Mechanisms to involve a broad spectrum of stakeholders, including the children themselves, parents, clinicians, and developers, in the design and evaluation processes.
4. Iterative Validation: Emphasis on continuous testing and validation of AI systems in real-world pediatric settings to ensure safety, accuracy, and ethical alignment.

### Lifecycle ethical integration

A distinctive attribute of the PEARL-AI framework is its focus on integrating ethical considerations into every phase of the AI lifecycle, including:

1. Problem Definition: Ensuring that the AI initiative addresses a genuine pediatric healthcare need without introducing unnecessary risk.
2. Data Collection and Preparation: Advocating for transparency, informed consent (tailored to the pediatric context), and equitable data representation.
3. Algorithm Development: Prioritizing fairness, explainability, and bias mitigation in model design.
4. Testing and Deployment: Instituting rigorous testing protocols to validate that AI tools perform reliably and safely in diverse pediatric populations.
5. Post-Deployment Monitoring: Establishing mechanisms for ongoing surveillance of AI systems to detect and rectify issues that may emerge over time.

The PEARL-AI framework emphasizes that AI in pediatric healthcare should not merely meet technical and clinical benchmarks but should actively protect and promote the best interests of children. By prioritizing safeguards through the AI lifecycle, the framework helps to ensure that the transformative potential of AI in child health is harnessed responsibly, with children’s rights and well-being placed at the forefront.

## Conclusion

This review article describes ethical principles and challenges in the use of AI in healthcare for children. Important AI ethical principles discussed include non- maleficence, beneficence, autonomy, justice and transferability, transparency and explainability, privacy, dependability, auditability, knowledge management, accountability, and trust. In the final section in this article, we provide recommendations for child-centered medical AI. We based our recommendations for child-centered AI on the policy guidance on AI for children by UNICEF, and we elaborated on these recommendations in the context of child health. We also introduced the Pediatrics EthicAl Recommendations List for AI (PEARL-AI) framework, that can be used by both AI developers and clinicians to ensure ethical AI-enabled systems in healthcare for children.
